# Recycling Technologies for Cathode Materials from Spent Lithium Iron Phosphate Batteries: An Overview

**DOI:** 10.3390/ma19040674

**Published:** 2026-02-10

**Authors:** Zhiwei Wang, Xin Chen, Lili Xing, Yurong Zhang, Mengjie Liu, Chengwei Zou, Wentianyu Zhang, Saifei Pan, Haojie Li, Xuetao Wang

**Affiliations:** 1 College of Vehicle and Traffic Engineering, Henan University of Science and Technology, Luoyang 471003, China; 2 Lomon Billions Group Co., Ltd., Jiaozuo 454191, China

**Keywords:** waste lithium iron phosphate batteries, battery failure mechanism, recycling of positive electrode materials

## Abstract

With the accelerated deployment of new energy vehicles and the global pursuit of carbon neutrality and carbon peaking goals, lithium iron phosphate (LFP) batteries have become a dominant technology in the energy storage market. The rapid expansion in their production and application has led to a surge in the number of spent LFP batteries, raising urgent concerns regarding resource recovery and environmental sustainability. This review provides a comprehensive overview of recycling technologies for spent LFP batteries, which are categorized into three major routes: (i) conventional metallurgical recycling, including pyrometallurgical and hydrometallurgical processes; (ii) direct regeneration strategies, such as electrochemical and solid-state approaches; and (iii) emerging green technologies, which leverage external fields or novel eco-friendly solvents to enhance recovery efficiency. The fundamental mechanisms, advantages, and limitations of each approach are systematically analyzed and compared. Finally, we also discuss current challenges and future directions for developing high-efficiency, low-cost, and environmentally benign recycling systems. These technological advances are expected to not only promote resource circularity and reduce ecological burdens but also provide a solid foundation for the sustainable evolution of the lithium-ion battery industry.

## 1. Introduction

**Figure 1:**
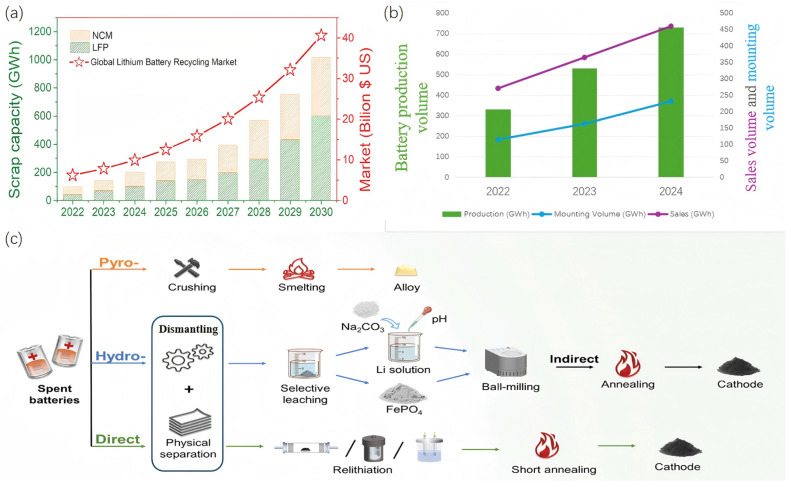
(**a**) The following report presents an estimate of the market size of global power batteries and energy storage batteries from 2022 to 2030 [9]. (**b**) Market Data of Lithium Iron Phosphate (LFP) Batteries in China Over the Past Three Years. (**c**) Lithium-ion Battery Production, Application, and Recycling Process [9].

Electric vehicles (EVs) and renewable energy systems are accelerating the global transition toward carbon neutrality, driving an unprecedented demand for high-performance energy storage devices. Among various lithium-ion battery chemistries, lithium iron phosphate (LiFePO_4_, LFP) batteries have emerged as a dominant technology, owing to their intrinsic safety, long cycle life, thermal stability, and cost-effectiveness. With their wide deployment in EVs, stationary energy storage, and grid-balancing applications, the global installed capacity of LFP batteries has reached several hundred gigawatt-hours annually and continues to grow at a double-digit rate. A comparison of data over the past three years is shown in Figure 1b. Such large-scale adoption reflects their indispensable role in supporting the decarbonization of transportation and power sectors worldwide [1,2,3].

However, the rapid expansion of LFP battery production and use has been accompanied by an equally rapid accumulation of end-of-life (EoL) batteries. As the first generation of large-scale LFP-based systems approaches retirement, a massive wave of spent batteries is expected in the coming years. This presents two major challenges: the efficient recovery of valuable materials such as lithium, phosphorus, and transition metals, and the mitigation of environmental hazards associated with improper disposal. In response, many regions—including the European Union (EU), the United States, Japan, and other major economies—have established comprehensive policies to promote battery recycling and resource circularity. The EU Battery Regulation (2022), for example, stipulates minimum recovery rates of 85% for lithium and 97% for critical metals, and requires manufacturers to adopt closed-loop recycling systems. Similarly, global initiatives such as the U.S. National Blueprint for Lithium Batteries (2021–2030) and Japan’s circular economy roadmap emphasize the creation of sustainable battery supply chains through efficient material recovery. Despite these policies, it is estimated that less than 5% of spent lithium-ion batteries are currently recycled worldwide, due to high transportation and collection costs, the technical complexity of disassembly, and limited industrial-scale recycling infrastructure [4]. From a technological standpoint, the recycling of LFP batteries presents unique challenges compared to nickel-rich or cobalt-based cathodes. The relatively low economic value of iron and phosphorus, the chemical stability of the olivine structure, and the complex composition of electrode binders and additives hinder efficient recovery. Nevertheless, rapid advances in materials science and green chemistry have catalyzed the development of a diverse set of recycling technologies, ranging from conventional metallurgical routes to innovative, environmentally friendly approaches.

Currently, three primary recycling routes dominate the industrial and academic landscape: hydrometallurgical, pyrometallurgical, and direct regeneration processes [5,6,7]. In particular, hydrometallurgical recycling offers high recovery efficiency, high product purity, and low operating temperature, but it requires intensive pre-treatment steps, consumes large volumes of acid and alkali reagents, and generates saline wastewater that demands secondary treatment. Pyrometallurgical recycling provides a simple and robust process with high throughput and tolerance to feedstock variability, yet it involves high energy consumption, emits harmful gases, and typically results in poor lithium recovery. In contrast, direct regeneration aims to restore the structure and electrochemical performance of cathode materials through targeted repair, re-lithiation, or surface modification. However, its scalability and compatibility with diverse waste streams remain key challenges [8]. Developing efficient, low-cost, and environmentally benign recycling systems for LFP batteries is thus of strategic importance for achieving a sustainable and circular battery economy. Beyond material recovery, advanced recycling can also reduce carbon emissions, alleviate resource dependence, and enhance the overall resilience of the global energy supply chain. In this context, this review provides a comprehensive and systematic overview of the state-of-the-art recycling technologies for spent LiFePO_4_ batteries. It classifies existing approaches into conventional metallurgical methods, direct regeneration strategies, and emerging green technologies that integrate external fields or novel eco-friendly solvents. Furthermore, it elucidates their underlying mechanisms, compares their advantages and limitations, and discusses future directions for realizing green, efficient, and industrial-scale recycling of LFP batteries. These technological advancements are expected to play a pivotal role in enabling sustainable energy storage systems and advancing the global low-carbon transition.

## 2. Summary of LFP Battery Failure Types and Mechanisms

**Figure 2:**
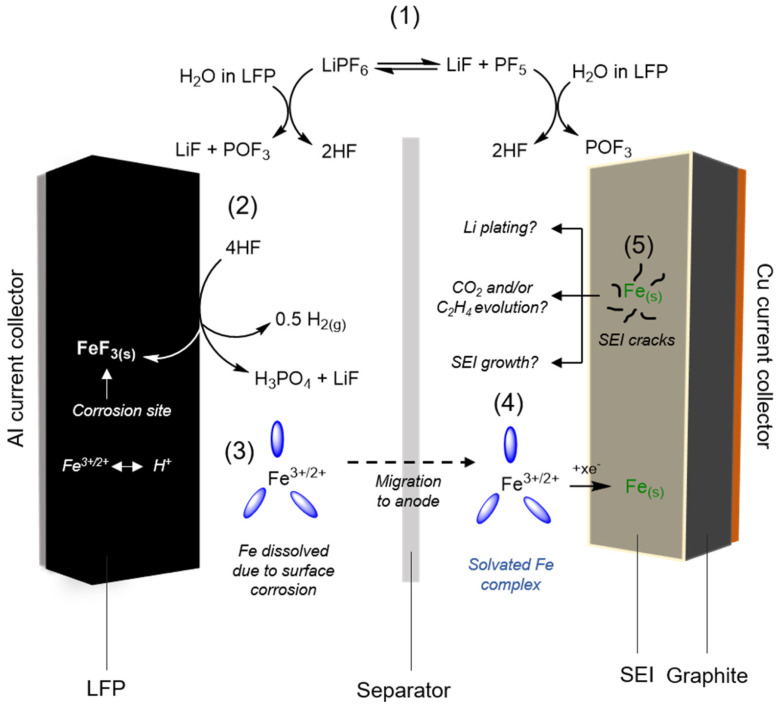
Battery deactivation mechanism (the purple oval represents dissolved iron ions) [28].

Lithium-ion batteries face battery failure or degradation issues during long-term cycling. Lithium loss, active material mass loss, and corrosion of the current collector and interface are the primary causes of failure [10]. The presence of impurities can also lead to battery failure [11,12,13]. Figure 2 illustrates the mechanism of battery deactivation.

The solid electrolyte interphase (SEI) forms on the anode, the cathode electrolyte interphase (CEI) forms on the cathode, and the growth of lithium dendrites all consume lithium within the battery [14]. During the initial charge cycle, the electrolyte encounters Li^+^ at the graphite surface and forms the SEI; at the cathode, the CEI forms. Each cycle disrupts these films, prompting their reformation. Each reformation consumes active lithium; the interface layers gradually thicken, increasing impedance and potentially triggering dendrite growth [15]. These dendrites not only extract more lithium from the cathode but may also bridge the electrodes, causing battery short circuits [16]. This process directly corresponds to the mechanism described in Step 5 of Figure 2 (the effect of metallic iron on the negative electrode SEI), where deposited metallic iron exacerbates SEI instability and the risk of dendrite growth.

The degradation of active materials stems from lattice expansion and contraction during each charge–discharge cycle. After hundreds of cycles, cracks begin to form at the edges of particles, exposing new surfaces that come into contact with the electrolyte. Subsequently, new SEI and CEI layers form on these exposed surfaces, consuming additional lithium. As cycling continues, these cracks gradually propagate. Simultaneously, localized lithium depletion oxidizes nearby Fe^2+^ to Fe^3+^, forming a thin FePO_4_ shell layer on the LiFePO_4_ particle surface [17,18]. Notably, FePO_4_ enables reversible lithium ion insertion/extraction in both non-aqueous and aqueous systems [19]; however, within the FePO_4_ shell formed under these degradation conditions, Fe^3+^ occupies the original lithium sites, disrupting the regular Li/Fe arrangement and blocking lithium ion diffusion pathways.

Capacity decay in lithium-ion batteries is often associated with reduced reversibility of lithium ion deintercalation/intercalation within the cathode material [20]. Once this occurs, residual lithium within the particles struggles to deintercalate or intercalate smoothly, weakening the particle’s structural framework and leading to diminished usable capacity [17,18]. This structural degradation of the cathode material and the formation of the FePO_4_ shell primarily correspond to Step 2 in Figure 2, where HF in the electrolyte reacts with the LFP surface, causing etching and iron dissolution.

When trace HF and free Fe^3+^ interact within the aggressive electrolyte, corrosion creates pinholes in the aluminum foil. Localized hotspots then accelerate electrolyte decomposition, causing nearby CEI swelling and self-amplifying damage. As pits deepen, aluminum ions leach out and deposit on the cathode particle surface, blocking electron pathways. Additional polarization intensifies, causing interfacial impedance to surge and exacerbating two further degradation mechanisms [21,22]. This chain reaction of aluminum foil corrosion can be traced back to Step 1 in Figure 2 (LiPF_6_ hydrolysis producing HF) and its subsequent effects, where HF serves as the key initiating factor for current collector corrosion.

Whatever the chemistry, LiFePO_4_ or ternary layered, graphite or silicon carbon anode, the cell dies for the same reason: the cell keeps losing lithium. The SEI and CEI thicken, lithium dendrites deposit, and every cycle adds fresh layers. Swelling and shrinking crack the grains; fresh cracks open new surfaces that instantly grow more films and devour more lithium. Fe^3+^ creeps into vacant lithium sites and blocks the paths [23]. Residual Fe_2_O_3_ and stray iron in the mix, plus high State of Charge (SOC) and heat, gnaw pits in the aluminum foil and force the electrolyte to break down. Lithium, active mass, and the conductive network all drain away together. To revive the cell, one must return the lithium, rebuild the interfaces, seal the cracks, and flush out the Fe^3+^ occupying the lithium sites. For degradation primarily driven by crystal structure defects, direct repair strategies such as electron beam irradiation demonstrate unique advantages. This technique enables precise control of irradiation dose to selectively degrade binders at low temperatures, facilitating efficient electrode delamination. Concurrently, it regulates the concentration of Li/Fe anti-site defects, inducing a transition in lithium ion diffusion pathways from one-dimensional to two-dimensional. This significantly enhances the efficiency of subsequent relithiation processes, achieving both structural repair and performance restoration of the material [24]. When the internal composition and valence states of a battery undergo changes, the restoration process must concurrently achieve both lithium replenishment and reduction. Two effective strategies are low-temperature direct regeneration using green deep eutectic solvents and synergistic repair with natural electron donors. Deep eutectic solvents (the Licl-ethylene glycol system) enable direct migration of lithium ions to material lithium vacancies at low temperatures (80 °C), while their reducing components simultaneously convert surface Fe^3+^ to Fe^2+^, achieving targeted lithium replenishment and defect repair [25]. Similarly, employing natural substances such as tea polyphenols as electron donors can simultaneously replenish lithium salts while efficiently converting the FePO_4_ phase into LiFePO_4_ and reducing Li–Fe anti-site defects [26]. For end-of-life lithium iron phosphate batteries where repair is economically unviable, a value-added upgrading strategy may be pursued. For instance, leveraging the stable structure and lattice of spent LFP materials, mild oxidation coupled with heterojunction engineering can transform them into high-performance electrocatalysts for oxygen evolution reactions. This direct conversion of retired batteries into high-value functional materials significantly enhances the economic viability of the recycling process [27].

## 3. Recycling Technology of Cathode Materials

Current lithium-ion battery cathode material recycling technologies can be summarized into three main approaches: traditional processes represented by pyrometallurgical and hydrometallurgical methods are mature but highly energy-intensive and polluting; direct regeneration strategies achieve low-energy closed-loop regeneration through lithium supplementation and structural repair, and are transitioning from laboratory research to commercial application; and novel recycling methods beyond traditional approaches utilize external field-assisted enhancement, novel green solvents, and plasma technology to strive for a future recycling landscape characterized by zero carbon emissions, high efficiency, and intelligence. Driven by policy initiatives and carbon neutrality constraints, direct regeneration and green enhancement technologies are poised to replace traditional high-energy-consuming processes, becoming the core technologies of the global lithium-ion battery recycling industry by around 2030 [29].

### 3.1. Traditional Recycling Technology

Traditional recycling technologies are primarily categorized into two main types: wet recycling and pyrometallurgical recycling. Among traditional recycling technologies, wet recycling of batteries requires pretreatment to reduce the difficulty and cost of subsequent metal leaching and material regeneration processes [30].

Pre-treatment technologies are primarily categorized into two main groups: physical methods and chemical methods. Chemical recycling technology refers to the addition of discharge electrolyte to consume the remaining battery power during the discharge process. However, the chemical method frequently causes metals to lose electrons, leading to oxidation and corrosion, which can easily result in electrolyte leakage and pollution of battery materials. Physical recycling techniques rely on the physical properties of different materials within spent LFP batteries to achieve separation. These methods are cost-effective and operationally simple. Physical treatment alone risks polluting the environment, so it serves only as the first step and must be paired with chemical methods to lift overall recovery [31,32,33]. Table 1 provides a detailed comparison of the advantages of various pre-treatment methods, with the most commonly employed techniques being crushing and magnetic separation. Crushing typically utilizes two mechanical force methods: shear crushing and impact crushing. The core process involves: first, safely discharging the battery and manually dismantling it to obtain the cathode plates, followed by applying either shear or impact crushing for varying durations. Research indicates that shear crushing significantly outperforms impact crushing in efficiency, achieving a dissociation rate of 42.08% within 5 s and reaching 69.29% after 40 s. In contrast, impact crushing exhibits a slower initiation of dissociation, with effective action concentrated between 5 and 20 s, ultimately yielding a dissociation rate of 62.68%. Both methods resulted in over-pulverization of the aluminum foil current collector, which becomes mixed with the fine powder, though its content could be controlled at a relatively low level [34]. Hu et al. [35] proposed employing high-intensity magnetic separation (HIMS) technology to recover LiFePO_4_ cathodes and graphite anodes from spent lithium iron phosphate (SLFP) batteries. Through a dry-process workflow of “discharging–crushing–magnetic separation–screening”, they found induction roller magnetic separation (IRMS) to yield optimal results for treating electrode fragments, achieving a LiFePO_4_ grade of 93.3% and a recovery rate of 98.7%. This method requires no chemical reagents, is environmentally sustainable, and offers low operational costs, presenting an efficient and readily industrializable new approach to physical separation.

During the pre-treatment process of lithium iron phosphate batteries, safety issues must be systematically managed. Although LFP batteries exhibit higher thermal stability compared to other lithium-ion chemistries, thermal runaway may still occur under overcharging conditions (110% SOC) or external high temperatures (>200 °C). Experiments indicate that when SOC exceeds 28%, thermal runaway may occur above approximately 200 °C due to anode reactions, with overcharging significantly reducing thermal stability [36]. Consequently, batteries must be fully discharged prior to dismantling to minimize residual energy and thermal runaway susceptibility [37,38]. The aqueous solution discharge method is recommended, particularly using NaCl solution, owing to its low cost, weak corrosivity, and ability to effectively absorb residual charge, thereby reducing short-circuit and overheating risks [39]. Lithium-ion battery electrolytes typically contain LiPF_6_ and organic carbonate solvents; LiPF_6_ readily decomposes upon contact with water or high temperatures, releasing toxic and corrosive gases such as HF and PF_5_. During wet crushing, LiPF_3_ primarily dissolves into water rather than being released through decomposition. The cooling and dissolving properties of the aqueous medium significantly suppress LiPF_6_ decomposition, reducing emissions of gaseous fluorine and phosphorus pollutants. Wet processes should be prioritized for dismantling and crushing operations, with circulation and monitoring ensured to promote LiPF_6_ dissolution and inhibit acid gas generation. Conversely, dry crushing or high-temperature processing may release toxic gases. Controling processing temperatures (maintaining water temperatures < 50 °C) and avoiding localized overheating can effectively suppress LiPF_6_ decomposition. Operators should routinely wear corrosion-resistant gloves, safety goggles, and respiratory protective equipment, whilst batteries require temperature and gas monitoring [40].

Pre-treatment technologies find extensive industrial application. Finland’s AkkuSer Oy employs a “dry process” comprising two-stage crushing followed by magnetic separation or cyclone degassing, achieving reagent-free, explosion-proof operations with high precious metal recovery rates; General Electric pursues a “mechanical separation and hydrometallurgy” approach, yielding copper, aluminum, iron, nickel, and lithium salts after acid leaching, followed by co-precipitation of ternary precursors with by-products including cobalt carbonate and lithium hyd roxide, graphite, aluminum foil, and black powder in a single step. The entire process requires no reagents and boasts an annual processing capacity exceeding 30,000 tons, making it both environmentally friendly and highly efficient [41].

**Table 1:** Physical treatment method of retired lithium iron phosphate battery.

Empirical Method	Distinguishing	Refs.
Crushing	Shear crushing method has large crushing ratio, large particle size, and good crushing effect on tough materials. Impact crushing method has high efficiency and low running cost.	[34,42]
Air Separation	Effective in material separation, but requires high uniformity of crushed products.	[43]
Eddy Current Separation	Effective in separation with strong adaptability.	[44,45]
Magnetic separation	Magnetic separation in batteries features high efficiency, cost-effectiveness, broad applicability, and customizability, enhancing material purity and safety.	[35,46]
Flotation	Adjustable settings; compatibility with various materials; high separation precision.	[47,48]

#### 3.1.1. Hydrometallurgical Recycling

Hydrometallurgical recycling is an effective method for recovering metals from the cathode materials of spent lithium–iron– phosphate (LFP) batteries. Hydrometallurgy dissolves metals in water-based reagents, then filters, purifies, extracts, and treats the wastewater [49]. It extracts metals from spent LFP cathodes with high yield and flexible chemistry, so leaching and separation are at its core. During the leaching process, the cathode materials are dissolved using acids or bases to extract metal components. Subsequently, the separation process involves techniques such as extraction and precipitation to obtain the desired metals and metal compounds [15,50,51]. Hydrometallurgical recycling is not only suitable for small and medium-scale recycling operations of spent lithium-ion batteries but is also particularly well-suited for treating LFP batteries.

**(1)**  **Acid leaching**

Acid dissolves valuable metals from spent LFP cathodes. Sulfuric, hydrochloric, and nitric acids are the usual choices [52,53]. In the acid, LFP breaks down into soluble iron and phosphate salts, while lithium enters the liquor. Sulfuric acid, combined with hydrogen peroxide, already yields high Li levels, and hydrochloric acid with the same oxidant achieves 96.31% under optimized conditions [54]. Table 2 lists the performance of each acid.

**Figure 3:**
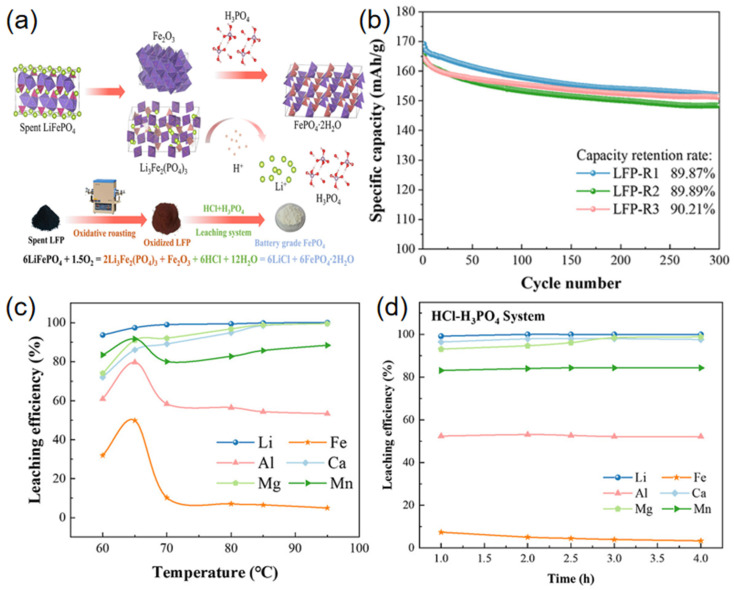
(**a**) Process flow (**b**) Battery cycle performance (**c**) Effect of different temperatures on elemental leaching rates (**d**) Effect of reaction time on elemental leaching rates (deep purple crystals: Fe_2_O_3_, deep red crystals: PO_4_, yellow spheres: Li) [61].

**Figure 4:**
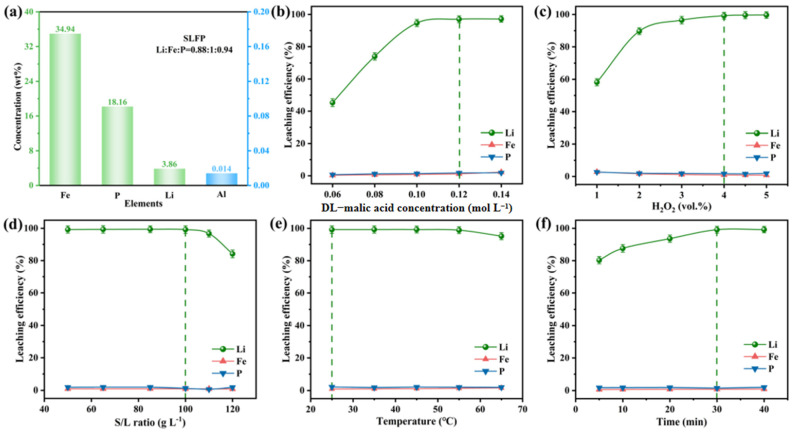
(**a**) Elemental content within the battery; (**b**) DL-malic acid dosage; (**c**) H_2_O_2_ concentration; (**d**) S/L ratio; (**e**) Temperature; (**f**) Effect of time on leaching rates of different elements (these green dotted lines indicate the optimum process conditions determined by the experiment) [62].

Luo et al. [61] proposed an innovative mixed acid leaching strategy employing hydrochloric acid and phosphoric acid to achieve selective lithium extraction and direct recovery of iron phosphate from spent lithium iron phosphate (LiFePO_4_) batteries. The process flow, as illustrated in Figure 3a, first employs oxidative roasting to separate materials, yielding feedstock with low impurity content. Subsequently, under optimized conditions (90 °C, liquid-to-solid ratio 5:1), mixed acid leaching with HCl–H_3_PO_4_ achieves both efficient lithium extraction (100% leaching rate, as shown in Figure 3c,d) and simultaneous synthesis of battery-grade FePO_4_·2H_2_O (iron loss rate merely 4.42%). The recovered iron phosphate successfully regenerates LiFePO_4_ cathode material with excellent electrochemical performance as a precursor, exhibiting a discharge capacity of 169.2 mAh/g at 0.5C and a capacity retention rate of 90.21% after 300 cycles, as shown in Figure 3b. This process avoids the use of oxidants, strong alkalis, and precipitants common in traditional hydrometallurgy, whilst enabling the recycling of phosphoric acid. It substantially reduces reagent consumption, energy expenditure, and secondary pollution, providing a sustainable solution for short-process, high-value closed-loop recycling of waste LFP batteries. Zhou et al. [62] developed an process for recycling waste LiFePO_4_ batteries based on malic acid. The method employs naturally degradable organic acids instead of traditional inorganic acid leaching, reducing the negative impact on the environment. Under optimized conditions, 99.12% Li is extracted, while less than 1% Fe is leached. Figure 4a shows the concentrations of different elements (Fe, P, Li, Al) in the regenerated battery. Figure 3b,c demonstrates the effects of different concentrations of hydrogen peroxide (H_2_O_2_) on leaching efficiency. Figure 4d–f illustrates the effects of solid–liquid ratio, temperature, and time on leaching efficiency. Compared with the traditional method, this process merits efficient lithium–iron separation, environmental friendliness, and economic efficiency. Biodegradable organic acid (DL-malic acid) instead of an inorganic acid is used together with an oxidant (H_2_O_2_) to selectively leach lithium, and innovatively recover P in the form of Li_3_PO_4_ by evaporation crystallization. It splits lithium from iron almost completely, cuts waste, and costs less than the former acid routes. The recycled products can be directly used to prepare high-performance recycled materials, making them the optimal choice for establishing a sustainable closed-loop recycling system. Li et al. [63] proposed a novel process for recycling metals from spent Lithium-Ion Battery (LIB) using a sulfuric acid–malonic acid leaching system that balances environmental sustainability and cost-effectiveness. Response surface methodology was employed to optimize process parameters. Under conditions of 70 °C for 81 min, with synergistic effects from 0.93 mol L^−1^ sulfuric acid, 0.85 mol L^−1^ malonic acid, a liquid-to-solid ratio of 61 mL g^−1^, and 30% H_2_O_2_, the lithium leaching rate reached 99.79%. The method works quickly and pulls out most metals, but it also floods the plant with acidic wastewater, burns through acid, and demands extra neutralizers. Cutting that waste and cost is now the main hurdle.

**(2)**  **Alkaline leaching**

Standard alkaline leaching solutions include NaOH, NaCO_3_, and NH_4_OH. In traditional processes, alkaline leaching is typically used to recover aluminum from cathode materials or to neutralize and precipitate acids in acid leaching solutions, and it is widely used in ternary batteries. Alkaline solutions have not yet been used as leaching agents for recovering waste LFP because iron cannot be leached into the solution under alkaline conditions. However, after material leaching, they can be used as iron precipitants [64]. The core of alkaline leaching technology lies in utilizing the selective dissolution and precipitation capabilities of OH^−^. In a strong alkaline environment, target metals (such as Li and Al) are preferentially leached out. At the same time, iron and phosphorus are induced to undergo in situ hydrolysis [64,65].

Yang et al. [66] proposed an innovative oxidative leaching process using sodium hydroxide for recycling spent LiFePO_4_. This method utilizes NaOH’s dual function as both a leaching agent and oxidant. Under optimal conditions of 2 mol/L NaOH, a liquid-to-solid ratio of 50:1, 50 °C, and 2 h, it achieved efficient and selective leaching of lithium (98.2%) and phosphorus (99.9%), while converting all iron into Fe_3_O_4_ residue. Kinetic studies indicated the process is controlled by chemical reaction. Mechanistic analysis revealed that OH^−^ oxidizes Fe(II) to Fe_3_O_4_ while being reduced to H_2_, and Na^+^ may intercalate into and disrupt the LiFePO_4_ structure. This process requires no additional oxidants or precipitants, directly yielding Li_3_PO_4_ from the concentrated leachate, offering advantages of low cost, simplicity, and environmental friendliness. Han et al. [67] proposed an alkali-enhanced Polyvinylidene fluoride (PVDF) cracking technology. Under conditions of NaOH concentration of 2.7 mol/L, solid–liquid ratio of 20 g/L, leaching time of 32 min, and leaching temperature of 34 °C, the aluminum leaching rate reached 92.6%. Subsequently, the alkali leaching residue was calcined at a lower temperature to catalyze PVDF cracking and convert it into harmless LiF. Treatment stripped 98.6% of the aluminum and left 97.5% of the lithium in hand. The rebuilt LFP delivered higher reversible capacity than the untreated powder; topping up lithium alone brought it back to battery grade. Zhang et al. [68] employed a sodium bisulfate-assisted roasting process under optimized conditions of 600 °C for one hour with a NaHSO_4_·H_2_O to LiFePO_4_ mass ratio of 1.5:1, achieving a high lithium leaching efficiency of 98.12% via water leaching of the resulting LiNaSO_4_. During roasting, the FeO_6_ and LiO_6_ octahedral units in LiFePO_4_ dissociate, enabling selective conversion of lithium into water-soluble LiNaSO_4_, while iron transforms into pure FePO_4_. The FePO_4_ recovered from the residue matched commercial-grade purity. Systematic experiments varying the mass ratio, roasting temperature, roasting time, and water leaching duration established the optimal parameters for maximal lithium recovery. The regenerated LFP material exhibited low polarization, comparable Li^+^ diffusion coefficients to commercial LFP, and excellent rate capability along with stable capacity retention over 200 cycles.

The process runs at room temperature and pressure, needs no extra heat, and keeps steps short and cheap. However, alkali leaching still hinges on concentration, solid-to-liquid ratio, temperature, and particle size, so that yields can swing widely. Laboratories combine alkali leaching with other recovery systems to improve recovery efficiency [66,67,69]. Optimizing leaching parameters and exploring new alkali leaching systems will be necessary to improve alkali leaching recovery rates while simultaneously reducing recovery costs.

**(3)**  **Bioleaching**

Microbes strip metals from spent LFP cathodes by gluing to the surface, then oxidizing or reducing the ions. Subsequently, these metals are released into the solution through oxidation and reduction reactions. Microorganisms produce biological leaching agents such as sulfuric acid or organic acids through metabolic activities. These substances can effectively dissolve metals in secondary resources like ores, end-of-life LIB, and Printed Circuit Board (PCB), as well as valuable metallic components in other electronic waste [70]. Commonly used bacteria are sulfur-oxidizing bacteria, iron-oxidizing bacteria, and Aspergillus niger strains [71,72].

Gu et al. [73] recovered Li from spent LiFePO_4_ batteries efficiently by bioleaching using an acidophilic bacterial consortium (ABC) enriched from acid mine drainage, dominated by Acidithiobacillus and Acidiphilium. They achieved a high recovery rate of 98% and confirmed that this method is more environmentally sustainable and cost-effective than traditional techniques through a carbon footprint assessment. Ghahreman et al. [74] conducted selective lithium leaching using the neutrophilic bacterium Thiobacillus under near-neutral pH conditions. At 30 °C and near-neutral pH conditions, lithium recovery reached approximately 65% (slurry density 15 g/L, leaching for 47 days). Under optimized conditions featuring lower slurry density (≤10 g/L), extended leaching duration (approximately 60 days), and continuous replenishment of bacterial cultures, lithium recovery increased to approximately 98%. Iron leaching remained consistently below 1%, demonstrating high leaching selectivity. Then, the recovered lithium is converted into Li_3_PO_4_, and the leachate is recycled as a culture medium. This process introduces an innovative LFP positive electrode material recycling method with good environmental prospects. The core of LIB bioleaching technology’s industrialization lies in its microbial strains simultaneously meeting the requirements of multi-metal toxicity tolerance, low-cost cultivation, and high-solids tolerance, while maintaining a metal leaching rate of 80–90% even under conditions of 100 g dry residue per liter of pulp [75].

The advantages of this technology lie in its environmental friendliness and low energy consumption. However, the efficiency of biological leaching is restricted by many factors, including the species of microorganisms, the growth environment, and the duration of response, which leads to their usually low recovery efficiency. At the same time, the cultivation and domestication process of microorganisms also increases the complexity and economic cost of the recovery process. Therefore, improving the recovery efficiency of bio-leaching and reducing the related costs are important problems that need to be solved in developing this technology.

#### 3.1.2. Pyrometallurgy

Pyrometallurgical recycling is a technology that uses high-temperature roasting, smelting, distillation, and molten salt to convert scrap metal, electronic waste, and other materials into reusable raw materials. Heat melts the oxides back to metal and drives off impurities in one step [76,77]. It handles large volumes, recovers most metals, and copes with mixed waste better than wet or mechanical routes [78].

**Figure 5:**
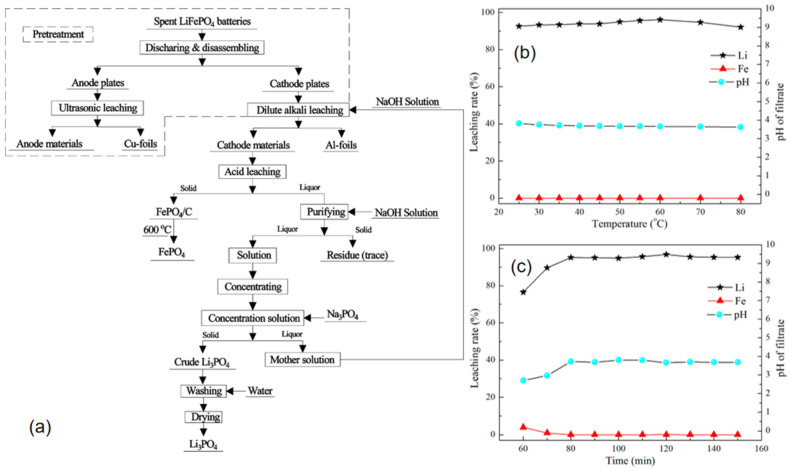
(**a**): Process flow diagram; (**b**,**c**): Effects of temperature and time on different metals (Dotted box: encompasses the entire ‘pretreatment’ process from waste batteries to electrode plate separation; solid arrow: indicates the sequential relationship of process steps and the flow direction of materials/solutions) [55].

Zhang et al. [79] discovered that under the influence of Na_2_CO_3_, LiFePO_4_ can be effectively decomposed via carbon thermal reduction below 600 °C, thereby achieving highly efficient lithium recovery with a recovery rate as high as 99.2%. The core principle lies in Na_2_CO_3_ acting as an activator, capable of breaking the thermodynamically stable olivine structure chemical bonds in LiFePO_4_. This overcomes the challenge that carbon cannot directly reduce LiFePO_4_, thereby enabling the reduction reaction to proceed. Following Na_2_CO_3_ activation, LiFePO_4_ is converted into products including Fe, NaLi_2_PO_4_, and LiNa_5_(PO_4_)_2_. Subsequent magnetic separation yields Li_3_PO_4_ or Li_2_CO_3_. Substituting the activator with NaOH still maintains a lithium recovery rate of 92.7%, wherein LiFePO_4_ primarily converts to Fe_3_O_4_ and identical lithium–sodium phosphates under oxidative conditions. This salt-assisted carbon thermal reduction–magnetic separation process combines high recovery rates, low energy consumption, and excellent scalability. Zhang et al. [68] mixed spent LFP cathode with NaHSO_4_ and fired the blend at 600 °C for one hour. After cooling, they poured the cake into water at 30 °C for an hour. The water pulled out 98% of the lithium while leaving Fe, Al, and P below 1% in solution. The solids—Li_3_PO_4_ and FePO_4_—went straight back into fresh LiFePO_4_. The rebuilt cathode delivered 162 mAh g^−1^ at 0.1 C, 120 mAh g^−1^ at 5 C, and still held 141 mAh g^−1^—97% of its initial capacity—after 200 cycles at 1 C. Qu et al. [80] roasted spent LFP with (NH_4_) _2_SO_4_ at 300 °C for ten minutes. Within five minutes, sulfur escaped as SO_2_, 98% of the lithium leached out as Li_2_SO_4_, and only 1.8% of the iron followed; clean FePO_4_ stayed behind. These short, acid-free salt roasts pull lithium back and rebuild high-rate LFP cheaply, yet the exact times and ratios still need fine-tuning. Li et al. [55] proposed a dual-system recovery process combining leaching and calcination for the efficient recovery of lithium, iron, and phosphorus from spent lithium iron phosphate battery cathode materials. Figure 5a illustrates the process flow diagram. Spent batteries undergo pretreatment with 0.3 M sulphuric acid and hydrogen peroxide, followed by selective leaching under optimized conditions (H_2_O_2_/Li molar ratio 2.07, H_2_SO_4_/Li molar ratio 0.57, 60 °C for 120 min), enabling lithium to be leached into solution at a high rate of 96.85%, while iron and phosphorus remain in the leaching residue as iron phosphate (iron leaching rate only 0.027%). Figure 5b,c illustrates the effects of time and temperature on different metals. The leachate underwent alkaline purification and concentration before sodium phosphate precipitation to recover lithium, yielding a lithium phosphate product with a lithium recovery rate of 95.56%. The leaching residue was calcined at 600 °C for 4 h to remove carbon residues, after which it was directly recovered as iron phosphate.

Pyrometallurgy demands furnace temperatures above 800 °C, so lithium evaporates and the LFP grains crack. Yields drop while the power bill climbs, eroding any green advantage. LFP waste contains few valuable metals at low prices, and the market price of finished LFP is generally lower than that of ternary materials, making pyrometallurgical lithium extraction even more challenging. The rigid olivine lattice needs extra heat to fall apart, pushing costs higher. High energy, high emissions, and thin returns leave pyrometallurgy with an uncertain future for LFP recycling.

### 3.2. Direct Regeneration Strategy Recycling Technology

#### 3.2.1. Solid Phase Regeneration Method

Solid-phase regeneration technology is a method for repairing and regenerating spent lithium-ion battery cathode materials through direct high-temperature heat treatment. This technology essentially involves a simple “supplementation-calcination” process: first, the recovered spent cathode active material is mixed with an appropriate amount of lithium source (such as Li_2_CO_3_, LiOH) to compensate for lithium loss during cycling at a stoichiometric ratio [81]. Subsequently, the mixture is calcined under an inert or controlled atmosphere to induce crystal structure rearrangement and regeneration, thereby restoring the material’s original crystalline structure and electrochemical performance [82].

Sun et al. [83] subjected cathode materials to thermal decomposition and separation, followed by sintering at 800 °C for 2 h in air to remove carbon coatings and conductive agents, yielding Fe_2_O_3_ and Li_3_Fe_2_(PO_4_)_3_. These were subsequently ball-milled with sucrose (20 wt%) and varying proportions of Li_2_CO_3_, pressed into discs, and regenerated via two-step sintering in an H_2_/Ar atmosphere. The sample containing 1.4 wt% Li_2_CO_3_ exhibited optimal performance, with the full cell maintaining over 80% capacity retention after 2000 cycles at 10 C. Guan et al. [84] proposed a method for regenerating LiFePO_4_ based on the Polyvinyl acetate (PVAc) alcoholysis reaction. Following pre-treatment of end-of-life batteries and NaOH washing to remove aluminum impurities, Li_2_CO_3_, FeC_2_O_4_, NH_4_H_2_PO_4_ and PVAc were added in a Li:Fe:P ratio of 1.05:1:1. The mixture was dispersed in a methanol-ammonia solution and regenerated via 6 h of pre-calcination at 350 °C followed by 12 h of main calcination at 650 °C under a nitrogen atmosphere. Samples incorporating 2.5 wt% PVAc exhibited optimal performance: initial discharge capacity reached 163.2 mA h g^−1^ (0.1 C), with a capacity retention rate of 97.08% after 100 cycles. Song et al. [85] studied the performance of high-temperature solid phase reaction after mixing newly synthesized LiFePO_4_ directly with waste material. When the mass ratio of the new and old materials was 3:7, and heated at 700 °C for 8 h, the resulting repair materials showed excellent electrochemical properties.

Solid-phase regeneration technology effectively restores the crystal structure and electrochemical properties of spent cathode materials, enabling their reuse. This regeneration process not only preserves the molecular structure of the cathode but also enhances material crystallinity and consistency, thereby directly improving its electrochemical performance. However, this method faces significant challenges: on one hand, the complex and inconsistent composition of waste materials makes precise elemental stoichiometric compensation extremely difficult; on the other hand, the process itself relies on prolonged high-temperature heat treatment, resulting in high energy consumption and the potential generation of dust and exhaust gases during processing, posing risks of secondary pollution. Therefore, to achieve high-capacity, long-cycle-life recycled materials, strict control over key process parameters—including raw material composition, processing temperature, duration, and atmosphere—is essential.

#### 3.2.2. Electrochemical Process

The electrochemical method extracts valuable metals from the cathode material of waste lithium iron phosphate batteries through electrochemical action. Currently, in electrochemical lithium extraction technology, ion exchange methods and membrane separation processes (such as electrodialysis) are commonly employed to recover lithium from liquid resources [86]. The electrochemical method crushes and screens the spent cathode, then electrochemically dissolves its metals; finally, pure metal is plated from the liquor in one electrolytic step. The electrochemical method has the advantages of simple operation and high recovery efficiency, and it is also a potential recovery method.

**Figure 6:**
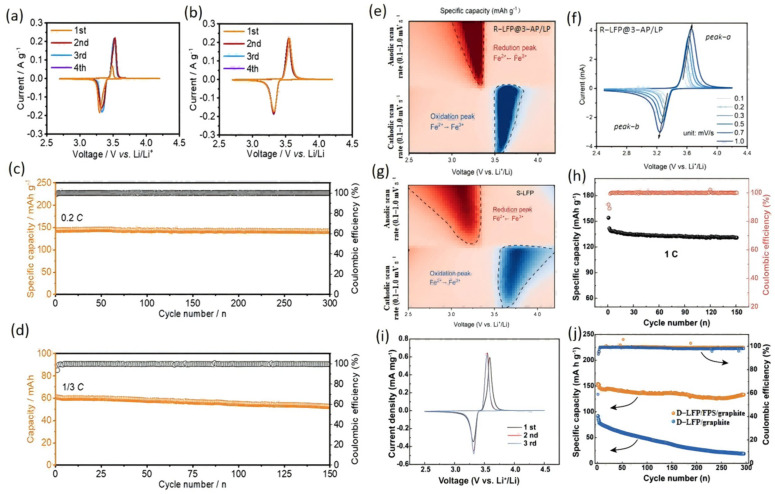
(**a**), Cyclic voltammetry curves of (**b**) spent lithium iron phosphate at 0.1 mV s^−1^. (**c**), Capacity versus cycle number and charge–discharge efficiency versus cycle number curves of recycled lithium iron phosphate at 0.2 C. (**d**), long-term cycling performance of recycled LiFePO_4_ at 1/3 C [89]. (**e**), Contour plots of specific capacity for recycled LiFePO_4_ illustrating the evolution of redox peaks across various cycle numbers and voltage ranges. (**f**), CV curves of recycled LiFePO_4_ at scan rates from 0.1 to 1.0 mV s^−1^. (**g**), Comparative contour plots tracking redox peak evolution in commercial LFP versus recycled during cycling [26]. (**i**) First three CV cycles of recycled LiFePO_4_ at 0.1 mV s^−1^. (**h**), Cycling performance of LFP batteries at 1 C rate after initial activation at 0.1 C. (**j**), Comparison of cycling stability between recycled and virgin batteries (arrows indicate which vertical axis corresponds to each line) [90].

Zhou et al. [87] proposed an environmentally friendly and economical electrochemical regeneration technique. The technology is used in water-based media, using an H-type electrolytic cell to build a low-cost battery system, where the cathode uses cheap zinc metal, and the positive electrode is the waste LiFePO_4_ material that needs to be recycled. During the discharge process, the lithium ion (Li^+^) is re-embedded into the old LiFePO_4_ to realize the regeneration of the material. Under the optimal conditions, the lithium content in the regenerated LiFePO_4_ can reach 3.81%, showing a good crystal structure and excellent electrochemical properties. Moreover, the regeneration process can be monitored in real time by Raman spectroscopy. Li et al. [88] leached lithium from spent LFP with 0.20 mol/L [Fe(CN) _6_]^3−^. The oxidant splits LiFePO_4_ into FePO_4_ and Li^+^, then the spent [Fe(CN) _6_]^4−^ is re-oxidized at the anode for reuse. At 99.8% Li recovery, the products are 99.9% LiOH and 99.97% FePO_4_. Wang et al. [89] proposed an efficient direct regeneration method for spent LiFePO_4_ cathode materials. Characterization revealed that after long-cycle battery operation, LiFePO_4_ transforms into Li_1-x_FePO_4_ due to lithium loss, forming FePO_4_ nanodomains within particles while preserving the olivine framework. Researchers employed a mild chemical lithium replenishment treatment using a lithium-aromatic solvent, successfully restoring the FePO_4_ phase to pure LiFePO_4_ while eliminating phase boundaries and preserving structural integrity. Voltammetric cycling tests demonstrated that the regenerated LiFePO_4_ exhibited fully restored oxidation peak intensity and symmetry compared to degraded material, with redox potentials matching those of fresh material, confirming complete electrochemical performance recovery (Figure 6a,b). The regenerated material exhibits electrochemical performance comparable to new material in both half-cell and full-cell tests, achieving a capacity of 152.5 mAh/g with excellent cycling stability, as shown in Figure 6c,d. This method offers advantages including low energy consumption, a simple process, and recyclable reagents, providing a new strategy for the green and efficient recovery of poly-anionic electrode materials. Liu et al. [26] employed a process where waste materials were oxidatively leached to obtain lithium-containing solutions, which were then used as the medium for electrochemical lithium replenishment, achieving recycling of the leachate. Their cyclic voltammetry curves exhibited two distinct pairs of Li^+^ insertion/extraction peaks at −0.13 V and −0.58 V in the initial electrolyte. Dilution of the electrolyte caused the reaction potential to increase, and the peaks disappeared under high-fold dilution (as shown in Figure 6f). Compared to Figure 6g, the CV curve of the recycled LiFePO_4_ exhibits greater symmetry with a narrower potential difference between redox peaks, indicating significantly enhanced electrochemical reversibility. This recycled cathode delivers a discharge capacity of 154.2 mAh g^−1^ at 0.5 C, maintaining 91.0% capacity retention after 300 cycles at 1 C. Fan et al. [90] proposed an in-situ electrochemical regeneration of spent LiFePO_4_ electrodes by employing a functionalized pre-lithiated separator coated with Li_2_C_2_O_4_/CMK-3 composite material, which decomposes during the initial charge cycle to supply additional Li^+^ ions. Cyclic voltammetry revealed that LFP maintains structural stability at the high voltage of 4.5 V without significant side reactions, as shown in Figure 6i. This decomposes during the initial charge to supply additional Li^+^ ions, enabling the regenerated full cell to recover a discharge specific capacity of 152.0 mAh g^−1^ at 0.05 C. After 292 cycles at 1 C, the capacity retention rate remains as high as 90.7%, as shown in Figure 6h,j, indicating excellent rate performance.

The electrochemical method’s advantage is that it can efficiently recover valuable metals in the cathode material of a waste lithium iron phosphate battery and has little impact on the environment. However, the electrochemical method needs to consume a large amount of electric energy and electrolytes, and the electrolysis process will produce a large amount of waste gas and waste liquid, which needs to be properly treated. Moreover, the process parameters of the electrochemical methods need to be strictly controlled to ensure the recovery efficiency and product quality.

### 3.3. New Recycling Technology That Surpasses Traditional Recycling Methods

#### 3.3.1. Mechanical Activation Method

The mechanical activation method refines the valuable metal particles in the cathode material of waste lithium iron phosphate batteries by mechanical action. It improves their reactivity to facilitate subsequent recovery and treatment. Mechanochemical processes, which utilize ball mills to drive chemical reactions, have been effectively applied in metal recovery from various waste streams. Examples include extracting indium from discarded liquid crystal displays, recovering lead from cathode ray tube funnel glass, and separating rare earth elements from phosphors [91]. The method is simple and energy-efficient.

The key steps in the mechanical activation method are mechanical activation and subsequent processing. The mechanical activation process involves placing the pretreated cathode material in the mechanical activation equipment for ball grinding or stirring treatment and refining the valuable metal particles through mechanical action. The subsequent treatment process involves chemical or physical treatment of the mechanically activated cathode material to separate the valuable metal from its compounds [92,93].

**Figure 7:**
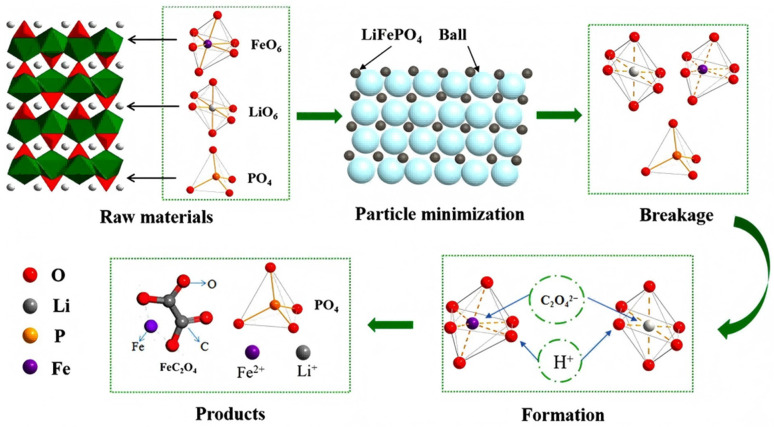
Products and mechanism of the mechanochemical process (the dark green crystals are FeO_6_, dark red crystals are PO_4_, white spheres are LiO_6,_ red corresponds to O, grey corresponds to Li, orange corresponds to P, purple corresponds to Fe) [95].

Fan et al. [94], used oxalic acid as grinding agent (oxalic acid and lithium iron phosphate cathode waste mass ratio of 1:1), and the ball after two h for water immersion. Test results showed that more than 99% of lithium and 6% iron was leaching. The main component of leaching residue was FeC_2_O_4_·2H_2_O, compared with the use of oxalic acid as leaching agent direct wet leaching, mechanical activation process significantly reduce the use of acid. Figure 7 illustrates the leaching reaction mechanism. Liu et al. [95] use Na and Li with similar outer electronic arrangement and coordination environment, using low-cost NaCl as abrasive agent and mechanical force to induce solid phase reaction, making Na isomorphism to replace Li in lithium iron phosphate. After mechanical activation, Na_2_CO_3_ solution was added to leach to obtain Li_2_CO_3_ precipitation, which also recycled NaCl. Li et al. [96] ball-milled the cathode powder with citric acid and H_2_O_2_ and leached 99.35% of the lithium; when citric acid and water were used instead, 97.82% Li and 95.62% Fe dissolved. The process is low-energy and uses no harsh reagents.

The advantage of the mechanical activation method is that it can improve the reactivity of valuable metals in the cathode material of waste lithium iron phosphate batteries and facilitate the subsequent recovery and treatment. However, mechanical activation requires a large amount of mechanical energy and produces a lot of dust and noise pollution. In addition, the recovery efficiency of the mechanical activation method is affected by various factors such as raw material composition and mechanical activation conditions.

#### 3.3.2. Ultrasonic Assisted Method

Ultrasound triggers cavitation: microbubbles form, oscillate, and collapse, producing high local temperature, pressure, shockwaves, and microjets. These accelerate mass transfer and fragment solids, enhancing reaction kinetics [97,98].

Paired with pretreatment, ultrasound cleaves organic binders and cleanly separates metal foils from active powders. The application of ultrasound during acid leaching reduces the leaching temperature and time required, accelerating and improving the metal leaching rate, lowering the activation energy of the reaction, and increasing the leaching efficiency. It can also be combined with hydrothermal methods to remove residual organics that affect remediation, which is beneficial for repairing channels blocked by cathode materials [99,100,101]. Zhou et al. [102] conducted experimental studies on the application of ultrasonic cavitation effects in recycling lithium iron phosphate (LiFePO_4_) batteries. They found that when the ultrasonic power was 80 W, the frequency was 40 kHz, the water temperature was 300 K, and the treatment time was 15 min, the removal efficiency of LiFePO_4_ could reach 77.7%. The recovered LiFePO_4_ powder also exhibited excellent electrochemical performance, indicating that the ultrasonic-assisted method is an efficient and green approach for recycling spent LiFePO_4_ batteries. Yang et al. [103] proposed a physical separation process combining ultrasonic-assisted alkali leaching with gas separation. Following discharge, positive and negative electrode sheets were separated. For positive electrode materials, treatment with dilute alkali (0.4 mol/L NaOH) combined with ultrasonication (70 kHz, 20 min, liquid-solid ratio 10 mL/g) was employed. Ultrasonic cavitation enhanced binder dissolution and interfacial delamination, achieving highly efficient separation of aluminum foil from LiFePO_4_ with a separation efficiency of 99.85%. For the negative electrode material, hammer crushing and general crushing are first performed, followed by classification based on particle size: copper foil with particle size > 0.25 mm can be directly recovered (copper grade 91.6%); the fraction with particle size < 0.106 mm yields high-purity graphite (grade 96.6%); while the intermediate size fraction (0.106–0.25 mm) undergoes gas separation. At an air velocity of 0.9 m/s, copper recovery reaches 87.2% with a grade of 84.6%. This entirely physical separation process significantly reduces chemical consumption and energy expenditure, offering an efficient and viable technical pathway for the large-scale, environmentally sound recycling of waste lithium-ion batteries.

Ultrasound is now ready for LFP recycling. Cavitation cracks the cathode lattice and speeds metal leaching with low energy and little waste. Tuning the transducer layout, pulsing the field, and scaling the reactor can ease the hurdles—modest throughput, uneven results, equipment drift and cost [104].

#### 3.3.3. Deep Eutectic Solvent Method

Deep Eutectic Solvent (DES) is a novel green solvent characterized by its low melting point, high dissolving capacity, and low toxicity. It is typically composed of a binary or ternary system formed by a hydrogen bond donor (such as polyols, urea, or carboxylic acids) and a hydrogen bond acceptor (such as quaternary ammonium salts, choline chloride). Currently, DES has been widely applied in battery recycling [105,106,107].

In the experiment, pretreated cathode material is mixed with DESs in a sealed glass bottle and subjected to an oil bath at different temperatures to facilitate complexation reactions, dissolving valuable metals efficiently. By adjusting the composition of DESs, reaction conditions can be optimized to enhance the selectivity of metal recovery. Finally, lithium and other metals are recovered through chemical precipitation [108,109,110].

**Figure 8:**
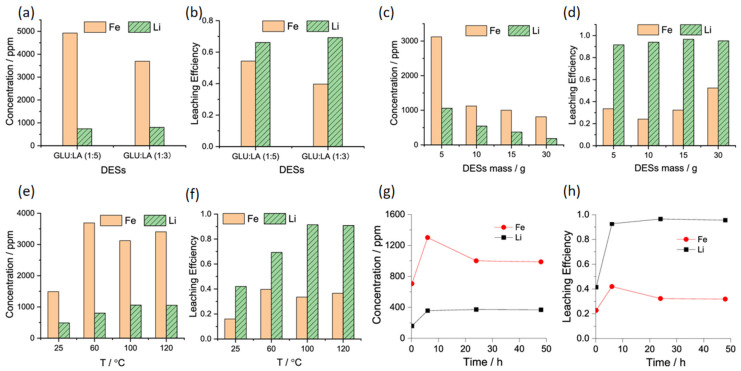
(**a**) Effect of glucose: lactic acid (GLU:LA) Molar Ratio on Fe and Li Concentration, (**b**) Effect of GLU:LA Molar Ratio on Fe and Li Leaching Efficiency, (**c**) Effect of DES Mass on Fe and Li Concentration, (**d**) Effect of DES Mass on Fe and Li Leaching Efficiency, (**e**) Effect of Temperature on Fe and Li Concentration, (**f**) Effect of Temperature on Leaching Efficiency, (**g**) Response time to changes in metal ion concentration, (**h**) Effect of reaction time on the dissolution rates of iron and lithium [112].

A.V. Kozhevnikova et al. [111] experimentally investigated the extraction and separation of valuable metals from spent lithium iron phosphate batteries using hydrophobic deep eutectic solvents (HDES) based on tributyl phosphate (TBP) and di(2-ethylhexyl) phosphoric acid (D2EHPA). Between 1 and 10 mol L^−1^ HCl, the extractant captured >99% Fe^3+^ at every tested acidity and >99% Al^3+^ at pH 1.4. Cu^2+^ stayed below 5% at low pH but rose to 50% at pH ≥ 1.9. Guided by these data, we devised a sequential route that lowers acidity stepwise to isolate Al^3+^, Cu^2+^, Fe^3+^, and Li^+^, and we set the conditions for strip solutions that regenerate the extractant and yield pure metal streams. Chen et al. [112] dissolved lithium selectively from spent LFP cathodes with a glucose–lactic acid deep eutectic solvent at mild temperature, achieving 96.5% Li recovery. Yield depends on solvent mass, time, temperature, and molar ratio. Figure 8a,b shows the lithium and iron concentrations and leaching efficiency at two different depths of discharge. It can be observed that the lithium concentration and leaching efficiency are significantly higher than those of iron. Figure 8c,d demonstrates the lithium and iron concentrations and leaching efficiency as the mass at depth of discharge increases. As the mass of DESs increases, lithium concentration and leaching efficiency increase, while iron concentration remains relatively stable. Figure 8e,f show that raising the temperature boosts lithium concentration and leaching efficiency, whereas iron concentration remains nearly constant. Figure 8g,h demonstrates the effect of time on lithium and iron concentrations and leaching efficiency (Figure 8h). Lithium concentration increased and stabilized with increasing time, while iron concentration showed little change. Regarding leaching efficiency, both lithium and iron improved with increasing time.

Although DESs are considered potential battery recycling lixiviants, their poor chemical stability, complex properties, high cost, and scalability challenges hinder practical application. The industrial sector is reluctant to adopt new DES-based recycling methods [113]. Additionally, research on the recycling of lithium iron phosphate batteries is limited. Mapping how DES components interact will clarify the extraction mechanism and guide the design of new systems.

### 3.4. Functionalised Recycling

Data from the International Energy Agency indicates that the current global lithium-ion battery recycling capacity stands at approximately 2 million tons annually. Meanwhile, the Organization for Economic Co-operation and Development notes that by 2030, over half of the world’s end-of-life lithium-ion batteries are projected to originate from electric vehicles, totaling around 1.6 million tons [114]. Confronted by this imminent scale of recycling, existing technologies reveal fundamental limitations. Currently, industrial pyrometallurgical and hydrometallurgical techniques, while capable of recovering high-value metals such as lithium and iron, suffer from high energy consumption, severe pollution (generating acidic waste liquids), and neglect of non-metallic component recovery. This results in resource wastage and environmental hazards [115,116]. This heavy-metal-priority recovery model essentially involves only selective extraction of certain metals from batteries, falling far short of achieving the goal of closed-loop utilization for all battery components. In existing processes, materials like carbon black and PVDF binders are often treated as worthless impurities and incinerated or discarded, failing to unlock their inherent value. “Functional recycling,” however, transcends simple disposal or removal. It focuses on leveraging the intrinsic properties of these components to transform them into functional materials with specific applications, thereby achieving value regeneration and enhancement [117].

Functional recycling follows two primary approaches: one involves direct reuse of components through physical disassembly, such as dismantling aluminum foil/copper foil current collectors or battery casings for use as structural parts in other products, or utilizing carbon black separated from spent electrodes as fillers in conductive coatings, conductive adhesives, and antistatic materials. This method offers advantages of operational simplicity and low energy consumption, but requires high purity and integrity of the waste materials [117,118]. The other approach involves upgrading waste materials into high-value-added materials through functional conversion. Zhou et al. [119] developed an iodine-mediated electrochemical extraction pathway that concurrently achieves lithium recovery and conversion of waste LFP into catalysts within the same liquid system. Leveraging iodine ion redox reactions, lithium is selectively leached with 93% recovery and precipitated as Li_2_CO_3_, while metallic zinc is cathodically deposited. The delithinated Fe_3_O_4_ retained carbon black and graphite conductive agents within its framework, serving directly as a 3D conductive network for OER catalysis. Mild alkaline leaching of PVDF generated fluorine-carbon defects, further enhancing oxygen evolution activity. This catalyst exhibited an overpotential of merely 250 mV at 10 mA cm^−2^, outperforming commercial RuO_2_. The entire process chain substantially reduces battery separation steps, enabling high-value utilization of waste batteries. Li et al. [120] achieved one-step electrode stripping at room temperature using methanol–citric acid as a dual-functional solvent, yielding intact aluminum foil without acid mist corrosion. The collected powder directly utilized the original 5 wt% PVDF from waste batteries as a fluorocarbon precursor. Following low-temperature solid-state sintering, the PVDF undergoes in-situ decomposition into fluorine-doped three-dimensional porous carbon networks. These networks tightly encapsulate the regenerated LiFePO_4_, while residual carbon black particles are welded into the carbon framework, forming continuous conductive pathways. The resulting cathode material exhibits a reversible capacity of 141.5 mAh g^−1^ at 1C, with only 0.4% capacity decay after 100 cycles. The aluminum foil, current collector, and conductive carbon are all retained, achieving one-step conversion of waste material into functional material. The aforementioned experiments demonstrate that the functionalization pathway eliminates strong acids and heavy metal-laden leachate, thereby reducing wastewater generation. Consequently, functionalized recycling significantly outperforms the conventional “acid leaching-resynthesis” route in terms of carbon footprint, resource consumption, and toxicity metrics.

In summary, the functionalized recycling of lithium iron phosphate not only breaks through the traditional linear model of “metal extraction–material resynthesis” but also unlocks its potential for reuse through structural repair and the imparting of new properties. While enhancing resource circulation efficiency, this approach significantly reduces environmental impact, providing both theoretical foundations and technical pathways for the sustainable management of waste lithium iron phosphate batteries. Future research should focus on the scale effects and interfacial evolution mechanisms of functional restoration. This will drive the transformation of LFP from “resource recovery” to “performance regeneration.”

### 3.5. Technical-Economic-Environmental Integrated Assessment

**Figure 9:**
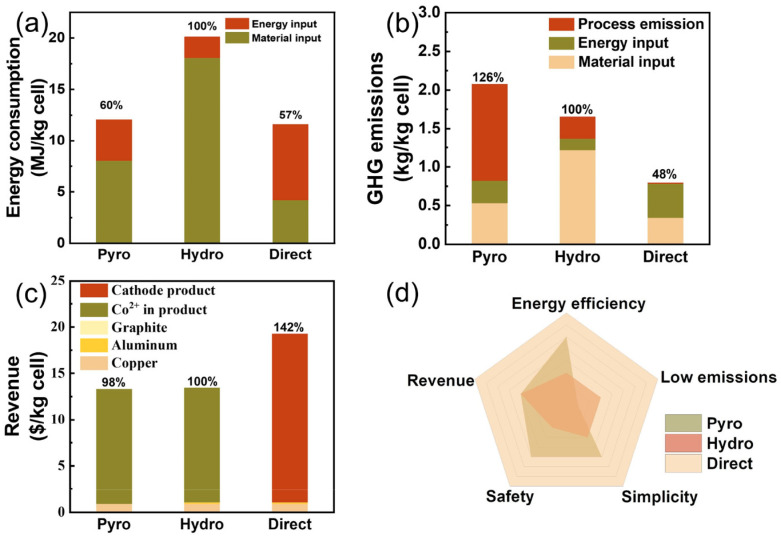
Illustrates the life cycle analysis results for pyrometallurgical, hydrometallurgical, and direct recycling methods. The comparison includes: (**a**) energy consumption, (**b**) greenhouse gas emissions, (**c**) potential revenue from recovered materials, and (**d**) a radar chart comparing various characteristics of the three recycling processes [121].

Regarding energy consumption, as shown in Figure 9a, hydrometallurgical recycling exhibits the highest overall energy demand due to the EverBatt model accounting for chemical reagent production. Recycling 1 kg of spent lithium-ion batteries requires approximately 21 megajoules (MJ) of energy, with roughly 18 MJ allocated to material inputs for chemical reagents (such as sulfuric acid, hydrochloric acid, hydrogen peroxide, etc.). Pyrometallurgical recycling exhibits significantly lower energy consumption than hydrometallurgical methods, primarily expended during high-temperature smelting. Recycling 1 kg of waste batteries requires approximately 13 MJ of energy. While its operational costs are relatively manageable due to reduced reliance on high-cost chemical reagents, it exhibits substantial carbon emission intensity. For LFP batteries, pyrometallurgical methods struggle to achieve efficient lithium recovery, with resource utilization rates below 40%. This further undermines their environmental and economic sustainability, making it difficult to meet future demands for high-value, low-carbon recycling. Direct recycling processes consume approximately 12 MJ of energy per kilogram of waste batteries, representing 57% of the energy required for hydrometallurgical recycling. While equipment energy consumption accounts for about 7.5 MJ in direct recycling—a relatively high proportion—the use of key chemical reagents as industrial byproducts eliminates additional energy consumption during their production, significantly reducing material energy consumption [121]. Wang et al. [122] further highlighted that in traditional hydrometallurgical recycling, reagents like NaOH and H_2_SO_4_ not only consume substantial energy but also generate significant wastewater treatment burdens. Their proposed approach involves “cross-system upgrading” the FePO_4_ component from low-value LFP batteries into sodium-ion battery systems while preserving high-value Li recovery pathways, achieving dual-track recycling through Li-Fe separation. This “dual-cycle” pathway significantly reduces energy consumption and chemical usage by eliminating Li replenishment and adopting sodium-based substitutes.

Regarding greenhouse gas emissions, as shown in Figure 9b, pyrometallurgical recycling processes emit approximately 2.1 kg of CO_2_ equivalent per kilogram of recycled waste batteries, representing the highest gas emissions. The hydrometallurgical recycling process emits approximately 1.6 kg CO_2_ equivalent per kg of recycled waste batteries, while the direct recycling process emits only 0.8 kg CO_2_ equivalent—just 48% of the hydrometallurgical method. Notably, the majority of greenhouse gas emissions across all three pathways stem from energy and material inputs, with minimal emissions generated during the recycling process itself [121]. Vinci et al. [123] indicate that within a life cycle assessment framework, the cascading utilization pathway (involving testing, sorting, disassembly, and reconfiguration of retired power batteries for downgraded applications in sectors with lower battery performance requirements) yields lower greenhouse gas emissions than traditional recycling processes. It is approximately 15 kg CO_2_ eq/kWh lower than pyrometallurgical recycling and about 11.9 kg CO_2_ eq/kWh lower than hydrometallurgical recycling. Wang et al. [122] found through LCA that their “dual-cycle” pathway reduces global warming potential by 30.2% compared to traditional linear recycling pathways and by 9.7% compared to closed-loop LFP regeneration pathways.

Regarding economic benefits, as shown in Figure 9c, the primary revenue sources for hydrometallurgical and pyrometallurgical processes stem from recovering high-value metals like cobalt and copper, with cobalt products accounting for over 90% of total revenue [121]. However, as battery chemistries evolve toward cobalt-free systems (LFP), the economic appeal of these traditional processes will gradually diminish. Wang et al. [124] also noted that LFP batteries, lacking high-value metals, yield significantly lower recycling economics than Nickel Cobalt Manganese Lithium-ion Batteries (NCM). Particularly in metal consumption reduction, LFP recycling achieves only about 30% resource savings, whereas NCM recycling exceeds 80%. In contrast, direct recycling processes yield a total profit of $3.9 per kilogram of recycled LFP waste, significantly higher than pyrometallurgical ($2.07) and hydrometallurgical ($2.44) methods, demonstrating substantial economic advantages for direct recycling in LFP battery recovery [125]. Moreover, the recycled LiFePO_4_ cathode material (approximately $19/kg) contributes the bulk of the value, with copper foil and other materials accounting for only a minor portion [121]. The high profitability of direct recycling stems from its near-100% lithium recovery rate and the direct usability of the recycled material, minimizing material loss and additional costs associated with re-synthesis [125].

Moreover, the radar chart (Figure 9d) demonstrates that the direct recycling process outperforms conventional methods across multiple dimensions. Beyond its low energy consumption, minimal emissions (11.9 kg CO_2_eq/kWh), and high economic returns, it features simpler operation and enhanced safety [121]. Utilizing fewer chemical reagents with lower toxicity, the direct recycling process offers greater operational feasibility and environmental friendliness for industrial applications [125].

Wang et al. [122] noted that their “dual-cycle” pathway outperforms traditional approaches across key environmental impact categories including resource scarcity, human health, and ecosystem quality. Notably, it reduces fossil resource scarcity by 29.1% compared to linear pathways and by 12.6% compared to closed-loop pathways. Wang et al. [124] also highlighted that cascading utilization of LFP batteries in energy storage systems significantly reduces net lifecycle impacts, particularly in fossil fuel consumption. Extending the cascading lifespan from 1 to 10 years enhances environmental benefits by 0.24–4.62 times. The literature also indicates that the direct recycling process currently has a Technology Readiness Level of 4. Although not yet industrialized at scale, some companies are conducting small-scale pilot projects, suggesting broad future development prospects [125].

The state of health (SOH) of a battery is the primary factor determining its post-retirement value and the appropriate recycling pathway. It is typically measured by the ratio of the battery’s current capacity to its initial capacity. The degradation of SOH signifies not only capacity loss but also indicates the evolution of the internal physicochemical structure [126]. Consequently, batteries in different states of health require distinct recycling methods: For batteries with higher SOH and relatively intact active material structures, direct recycling methods—sensitive to process conditions but requiring single-component feedstocks—are more suitable. Conversely, batteries with lower SOH and severe structural damage benefit from extraction processes such as hydrometallurgy or pyrometallurgy. In terms of industrial maturity, hydrometallurgy currently represents the most established and widely applied industrial method, particularly suited to feedstocks with a certain range of compositional variability, though leaching purity remains susceptible to impurities. Pyrometallurgy holds industrial application value under specific conditions, yet exhibits low adaptability to mixed feedstocks and may readily cause elemental alloying or slag entrapment. From an environmental perspective, direct recycling technologies align most closely with green development requirements due to their minimal chemical reagent consumption and lowest secondary pollution. Although currently experimental, technological advancements and cost reductions are anticipated to achieve industrial breakthroughs within the coming years.

It is worth noting that beyond the scaled-up pyrometallurgical, hydrometallurgical, and direct recycling routes mentioned above, a number of emerging recycling strategies have emerged in recent years. These include mechanochemical coupled selective leaching, deep eutectic solvent recovery, ultrasonic-assisted recovery, and functional recycling. Given that these processes remain in laboratory or early experimental stages, publicly available data exhibits significant gaps in mass balance, energy consumption benchmarks, and life cycle boundary definitions. This makes it challenging to conduct comparable technical-economic-environmental assessments against mature routes. Therefore, this paper intentionally excludes them from the comprehensive assessment phase, not to dismiss their potential advantages. This section is deliberately reserved as a future research direction, aiming to incorporate them into the reassessment framework once the data system is refined, thereby enabling dynamic updates to the integrated technical-economic-environmental evaluation of recycling technologies.

## 4. Conclusions and Future Prospects

This review has systematically examined the representative recycling approaches for spent lithium iron phosphate (LFP) batteries, highlighting their respective strengths and limitations. Each recycling route, including hydrometallurgical, pyrometallurgical, direct regeneration, electrochemical, mechanical activation, and ultrasonic-assisted recovery, offers unique advantages under specific conditions, yet all face persistent technical, environmental, and economic challenges. Building on these insights, future work should focus on targeted improvements across multiple levels.

1. Hydrometallurgical methods should leverage selective, non-corrosive solvents and novel catalysts to enhance leaching efficiency while reducing reagent consumption and wastewater generation. High-temperature solid-state and direct regeneration technologies can be optimized through lower-temperature calcination, shorter dwell times, or hybrid thermal–chemical strategies to decrease energy requirements and emissions. Electrochemical recovery could benefit from miniaturized, modular cells and reduced electrolyte volumes, enhancing scalability and energy efficiency. Mechanical activation techniques should prioritize energy-efficient milling, dust suppression, and noise reduction, while maintaining enhanced metal reactivity. Ultrasonic-assisted recycling methods, though promising in efficiency and material purity, require cost-effective reactor design and automation for industrial-scale application.
2. At the industrial level, comprehensive end-of-pipe treatments, including advanced wastewater neutralization, off-gas scrubbing, and solid residue management, will be essential to meet environmental regulations and achieve zero-waste targets. Standardization of process parameters, battery sorting, and cathode characterization will further enhance recovery yields and product consistency.
3. From a policy and supply-chain perspective, governments should implement mandatory life-cycle tracking, provide tiered subsidies for challenging chemistries, and foster public-private partnerships to share recycling infrastructure. International collaboration and data-sharing platforms could harmonize standards, enable cross-border material circulation, and accelerate the establishment of closed-loop supply chains.

Against the backdrop of the dual carbon goals, the continuous innovation of battery recycling methods holds significant strategic importance. Looking ahead to the future of battery recycling technology, future recovery processes will evolve towards multi-technology coupling and process optimization. For instance, hydrometallurgy can be combined with mechanical activation pretreatment to enhance the selective leaching rate of lithium; direct recycling processes may incorporate ultrasonic-assisted technology to improve the uniformity and efficiency of material recovery. Through the integration of artificial intelligence and big data technologies, a digital system spanning the entire battery lifecycle—from production and use to recycling—will be established. Real-time monitoring of battery health will predict optimal decommissioning timings and recycling value, enabling precise recycling and management. During processing, machine learning will optimize parameters to enhance product consistency, propelling battery recycling towards intelligent development. Consequently, advancements in battery recycling technology not only constitute a vital safeguard for resource security but also represent an indispensable key component in achieving carbon neutrality.

## Figures and Tables

**Table 2:** Effect of Different Acid Leaching Methods on Metal Leaching Rates from Cathodes of Spent Lithium–Iron–Phosphate Batteries.

Solvent	Experimental Conditions	Leaching Efficiency	Product	Ref.
H_2_SO_4_ + H_2_O_2_	0.3 M/L H_2_SO_4_, H_2_O_2_/Li = 2.07, H_2_SO_4_/Li = 0.57, T = 60 °C, t = 2 h	Li 96.85%, Fe 0.027%	Li_2_PO_4_, FePO_4_	[55]
H_2_SO_4_ + H_2_O_2_	0.3 M/L H_2_SO_4_, H_2_O_2_/Li = 1.03, H_2_SO_4_/Li = 0.52, T = 25 °C, t = 1.5 h	Li 99.97%, Fe 0.021%	Li_2_CO_3_, FePO_4_	[56]
H_2_SO_4_	2.5 mol/L H_2_SO_4_, L/S = 10 mL/g, T = 60 °C, t = 4 h	Li 97%, Fe 98%	Li_2_CO_3_, FePO_4_	[57]
H_2_SO_4_	H_2_SO_4_/Fe = 1.1:1, L/S = 4:1, T = 25 °C, t = 1.5 h	Li 97.5%, Fe 96.5%	FePO_4_·2H_2_O	[58]
H_2_SO_4_ + air	P(air) = 0.4 Mpa H_2_SO_4_/Li = 0.5, L/S = 20 mL/g, T = 90 °C, t = 5 h	Li 98.9%, Fe 0.41%	Li_2_CO_3_, FePO_4_	[52]
HCl + H_2_O_2_	6.5 mol/L HCl, L/S = 5 mL/g, T = 60 °C, t = 2 h	Li 92.15%, Fe 91.73%	Li_3_PO_4_	[53]
HCl + NaClO	HCl/Li = 0.8, NaClO/Li = 1, t = 1 h	Li 99%, Fe 91.73%	Li_2_CO_3_, FePO_4_	[51]
H_3_PO_4_	0.5 mol/L H_3_PO_4_, T = 95 °C, t = 12 h	Li 99.2%, Fe 97.68%	LiH_2_PO_4_, FePO_4_·x H_2_O	[59]
CO_2_ + H_2_O	P(CO_2_) = 2 MPa, S/L = 100 g/L, t = 3 h	Li 96.8%, Fe 0.22%	Li_2_CO_3_, FePO_4_	[60]

## Data Availability

No new data were created or analyzed in this study. Data sharing is not applicable to this article.

## Abbreviations

The following abbreviations are used in this manuscript:

EVs	Electric vehicles
LFP	LiFePO_4_
EoL	end-of-life
EU	European Union
U.S.	United States
SEI	Solid Electrolyte Interphase
CEI	Cathode Electrolyte Interphase
SOC	State of Charge
HIMS	high-intensity magnetic separation
IRMS	induction roller magnetic separation
LIB	Lithium-Ion Batteries
PVDF	Polyvinylidene fluoride
PCB	Printed Circuit Board
ABC	acidophilic bacterial consortium
PVAc	Polyvinyl acetate
XRD	X-ray Diffraction
SEM	Scanning Electron Microscopy
DES	Deep Eutectic Solvent
HDES	hydrophobic deep eutectic solvents
TBP	tributyl phosphate
D2EHPA	di(2-ethylhexyl) phosphoric acid
GLU	glucose
LA	lactic acid
MJ	megajoules
NCM	Nickel Cobalt Manganese Lithium-ion Batteries
SOH	state of health
SLFP	spent lithium iron phosphate
